# Filter cigarette smoking and lung cancer risk; a hospital-based case–control study in Japan

**DOI:** 10.1038/sj.bjc.6601565

**Published:** 2004-02-03

**Authors:** T Marugame, T Sobue, T Nakayama, T Suzuki, H Kuniyoshi, K Sunagawa, K Genka, N Nishizawa, S Natsukawa, O Kuwahara, E Tsubura

**Affiliations:** 1 Statistics and Cancer Control Division, Research Center for Cancer Prevention and Screening, National Cancer Center, 5-1-1 Tsukiji, Chuo-ku, Tokyo 104-0045, Japan; 2 Department of Cancer Control and Statistics, Osaka Medical Center for Cancer and Cardiovascular Diseases, 1-3-3 Nakamichi, Higashinari-ku, Osaka 537-8511, Japan; 3 Osaka Anti-Lung Cancer Association, 4-6-5 Doshomachi, Chuo-ku, Osaka 541-0045, Japan; 4 Miyako Public Center, 476 Higashinakasone, Aza, Hirara, Okinawa 906-0067, Japan; 5 Okinawa Prefectural Miyako Hospital, 801 Higashinakasone, Aza, Hirara, Okinawa 906-0067, Japan; 6 National Okinawa Hospital, 3-20-14 Ganeko Ginowan Okinawa 901-2214, Japan; 7 Saku Central Hospital, 197 Usuda, Minamisaku, Nagano 384-0301, Japan

**Keywords:** lung cancer, histological type, filter and nonfilter cigarettes, hospital-based case–control study, Japan

## Abstract

Recent changes in the histology of lung cancer, namely a relative increase of adenocarcinoma compared to squamous cell carcinoma, might be due to a temporal shift from nonfilter to filter cigarettes. To investigate the association between type of cigarette and lung cancer by histological type, we conducted a case–control study in Japan, comprising 356 histologically confirmed lung cancer cases and 162 controls of male current smokers, who provided complete smoking histories. Overall, logistic regression analysis after controlling for age and prefecture revealed decreased risk, as shown by adjusted odds ratios, for both squamous cell carcinoma and adenocarcinoma among lifelong filter-exclusive smokers as compared to nonfilter or mixed smokers. This decrease was greater for squamous cell carcinoma than for adenocarcinoma. Among men under 54 years, filter-exclusive smokers displayed increased risk of adenocarcinoma, but decreased risk of squamous cell carcinoma. The recent shift in histology from squamous cell carcinoma to adenocarcinoma, particularly among younger smokers, might be due to changes in cigarette type. However, among subjects aged 65 years or more, no differences in histological type appeared related to type of cigarette smoked, implying that other factors are associated with increases in adenocarcinoma among older Japanese population.

Recently, the overall incidence of lung cancer has increased in Japan. However, incidence by histological type has shown a changing pattern. A relative increase in incidence of adenocarcinoma (AC), as compared to squamous cell carcinoma (SCC), has been observed, particularly for the younger age group (Tanaka *et al*, 1988; Yoshimi *et al*, 2003). While the same trends have been demonstrated in Western countries (Levi *et al*, 1997; Russo *et al*, 1997; Skuladottir *et al*, 2000; Janssen-Heijnen *et al*, 2001), AC accounts for a larger proportion of all lung cancer in Japan (Parkin *et al*, 1992). These relative increases in AC do not appear attributable to changes in pathological diagnosis alone (Charloux *et al*, 1997).

Changes in the composition of cigarettes, such as content of tar and nicotine, might influence lung cancer trends. The market share held by high-tar nonfilter cigarettes was almost completely taken over by low-tar filter cigarettes in the 1960s in both Japan and Western countries (Wynder *et al*, 1991). The links between changes in histology of lung cancer and type of cigarettes have led to the hypothesis that the type of cigarette, that is, filter or nonfilter, is associated with changing histological patterns of lung cancer. Several epidemiological studies have found that the effect of low-tar filter cigarettes on lung cancer risk differs according to histological type of tumour (Wynder and Kabat, 1988; Stellman *et al*, 1997). However, to date, no studies have examined possible relationships between type of cigarette and lung cancer risk by histological type in Asian populations. The present study explored the relationship between type of cigarettes smoked and lung cancer histology in Japan, focusing on differences between SCC and AC, by means of a multicentre, hospital-based case–control study.

## MATERIALS AND METHODS

A multicentre, hospital-based case–control study was conducted in 17 hospitals that participated in the Osaka Anti-Lung Cancer Association in Osaka prefecture, two hospitals in Okinawa prefecture, and one hospital in Nagano prefecture in Japan. In participating hospitals, patients were recruited from all lung cancer wards, in addition to one or more wards for other diseases. Study subjects comprised patients who were newly admitted to the participating hospitals from January 1996 to December 1998. A total of 1324 patients (945 men and 379 women) were admitted with newly diagnosed lung cancer. All lung cancer cases were confirmed microscopically. Controls comprised 3600 patients (2169 men and 1431 women) who were admitted to the same hospitals during the same period with diseases other than lung cancer. Of the 3600 controls, 2348 patients with diseases related to smoking were excluded, that is, no patients with respiratory tuberculosis (ICD-10: A15, 16, 19, B90), respiratory infection (A31), neoplasm (C00-D48), inguinal hernia (K40), ischaemic heart disease (I20-I25), subarachnoid haemorrhage (I60), arterial disease (I70-I73), respiratory disease (J00-J99), peptic ulcer (K25-K27), or respiratory symptoms (R04, R06, R09) were included in the study. After further exclusion of subjects ⩽39 or ⩾80 years (65 cases and 286 controls) and subjects who did not provide complete information on current smoking habits (104 cases and 86 controls), 1115 lung cancer cases and 880 controls remained for analysis.

Among the 880 controls, distribution of diagnoses was as follows: 19% ear and mastoid (H60-H95); 16% digestive system (K00-K93); 12% nervous system (G00-G99); 9% circulatory system (I00-I99); 9% endocrine, nutritional and metabolic (E50-90); 8% symptoms, signs, and abnormal clinical and laboratory findings not elsewhere classified (R00-R99); 5% infectious and parasitic (A00-B99); 5% musculoskeletal system and connective tissue (M00-M99); 4% genitourinary system (N00-N99); 4% injury, poisoning, and certain other consequences of external causes (S00-T98); 3% blood and blood-forming organs and certain disorders involving the immune mechanisms (D50-D89); 3% skin and subcutaneous tissue (L00-L99), 3% congenital malformations, deformations, and chromosomal abnormalities (Q00-Q99) and less than 2% for other categories.

Information on smoking history and other lifestyle factors was obtained by means of a self-administered questionnaire completed during admission. Current smoking status was confirmed using a closed question. Detailed smoking histories were then obtained from current and former smokers by means of open questions regarding ages at which smoking habits changed substantially; information on number of cigarettes smoked per day and type of cigarettes (filter, nonfilter, or others) was requested for each period.

To investigate associations between type of cigarette (filter/nonfilter) and histological type of lung cancer, we further examined male current smokers (356 cases and 162 controls) for whom complete smoking histories regarding filter/nonfilter cigarettes were available. Owing to the small number of female current smokers with complete smoking histories, this analysis was restricted to male current smokers. Duration of nonfilter or filter use was calculated separately, based on the history of smoking. Current smokers were categorised into two groups: ‘filter-exclusive smokers’ comprised men who were lifetime smokers of filter cigarettes; ‘mixed or nonfilter smokers’ were men who had smoked nonfilter cigarettes at some point. Any history of cigarette use before 1957, when filter cigarettes first became commercially available in Japan, was regarded as nonfilter cigarette use. The mean number of cigarettes per day was defined as the weighted mean of each average number of filter and nonfilter cigarettes smoked per day. Total duration was defined as the sum of durations of filter and nonfilter smoking. Subjects who reported smoking other types of cigarettes were excluded from the analysis.

Odds ratios (ORs) and 95% confidence intervals (CIs) were calculated using unconditional logistic regression analysis in order to estimate the risk of lung cancer by histological type. Statistical adjustment was made for age (continuous variable) and prefecture (three categories). Adjusted ORs in relation to type of cigarettes were presented with and without adjustment for mean number of cigarettes smoked per day (continuous variable). All statistical computations were performed using PC-SAS (SAS Institute Inc., Cary, NC, USA).

## RESULTS

Prevalences of SCC, AC, small cell carcinoma, large cell carcinoma, and unknown histology were 34.3, 44.0, 11.3, 3.9 and 6.4% for men and 8.9, 75.6, 7.6, 2.8, and 5.1% for women, respectively.

Current cigarette smoking was associated with increased risk of overall lung cancer, SCC and AC; adjusted ORs of current smokers as compared to nonsmokers were 4.56 (95% CI: 3.00–6.94) for all lung cancers, 24.5 (95% CI: 7.39–80.9) for SCC, and 2.56 (95% CI: 1.61–4.07) for AC, respectively, for men, and 2.29 (95% CI: 1.44–3.64) for all lung cancers, 10.9 (95% CI: 3.99–30.0) for SCC, and 1.48 (95% CI: 0.87–2.51) for AC, respectively, for women.

Adjusted ORs for lung cancer in relation to duration of smoking and number of cigarettes per day are presented by sex in Tables 1

**Table 1:** Adjusted odds ratios (ORs) for lung cancer associated with cigarette smoking by histological type among men

		**Cases**					
	**Controls**	**Squamous cell carcinoma**		**Adenocarcinoma**		**All lung cancers**	
**Smoking status**	**No.**	**No.**	**Adjusted**^a^ OR (95% CI)	**No.**	**Adjusted**^a^ OR (95% CI)	**No.**	**Adjuste**d^a^ OR (95% CI)
Nonsmoker	90	3	1.00 (reference)	30	1.00 (reference)	40	1.00 (reference)
Past smoker	161	87	13.9 (3.16–61.0)	120	1.95 (1.09–3.50)	246	2.46 (1.47–4.12)
Current smoker							
*Duration of smoking in years*							
1–20	13	2	5.65 (0.80–39.9)	6	1.56 (0.53–4.62)	9	1.85 (0.71–4.85)
21–39	137	52	17.4 (5.08–59.9)	94	2.38 (1.42–3.99)	189	3.74 (2.37–5.90)
40+	90	144	29.8 (8.96–89.4)	119	2.89 (1.70–4.90)	355	6.02 (3.76–9.62)
*Number of cigarettes per day*^b^							
1–20	104	69	3.00 (1.61–5.59)	84	1.67 (1.05–2.65)	197	1.94 (1.31–2.87)
21–39	88	73	7.11 (3.74–13.5)	80	2.23 (1.39–3.59)	210	3.38 (2.67–5.05)
40+	37	43	12.5 (5.88–26.6)	43	3.01 (1.69–5.37)	110	4.61 (2.80–7.57)

a Adjusted for age and prefecture.

b In total, 36 cases and 11 controls for current smokers were not included because of incomplete data. CI=confidence interval.

and 2

**Table 2:** Adjusted odds ratios (ORs) for lung cancer associated with cigarette smoking by histological type among women

		**Cases**					
	**Controls**	**Squamous cell carcinoma**		**Adenocarcinoma**		**All lung cancers**	
**Smoking status**	**No.**	**No.**	**Adjusted**^a^ OR (95% CI)	**No.**	**Adjusted**^a^ OR (95% CI)	**No.**	**Adjusted**^a^ OR (95% CI)
Nonsmoker	320	10	1.00 (reference)	195	1.00 (reference)	231	1.00 (reference)
Past smoker	28	8	9.56 (2.73–33.4)	14	0.54 (0.23–1.26)	29	0.93 (0.47–1.81)
Current Smoker							
*Duration of smoking in years*							
1–20	16	1	2.48 (0.28–21.8)	4	0.48 (0.15–1.50)	6	0.63 (0.23–1.68)
21–39	23	6	12.5 (3.77–41.5)	19	1.85 (0.95–3.63)	32	2.61 (1.44–4.74)
40+	2	3	40.2 (5.71–282.7)	7	4.26 (0.87–20.9)	18	9.34 (2.13–41.1)
*Number of cigarettes per day*^b^							
1–20	33	9	12.3 (4.34–35.0)	22	1.31 (0.72–2.37)	40	1.98 (1.18–3.32)
21+	6	1	7.54 (0.75–75.8)	7	3.09 (0.97–9.86)	13	4.37 (1.57–12.2)

a Adjusted for age and prefecture.

b In total, three case and two controls for current smokers were not included because of incomplete data. CI=confidence interval.

. For men, adjusted ORs among current smokers as compared to lifelong nonsmokers increased with longer duration of smoking and increasing number of cigarettes per day, irrespective of histological type of lung cancer (Table 1). The adjusted OR for SCC was much higher than that for AC. Male former smokers displayed an approximately 14-fold increase in risk of SCC, whereas elevation in the adjusted OR for AC was two-fold.

Similarly, in women, increasing risk regardless of lung cancer histology (as indicated by adjusted OR) with increasing intensity of smoking was observed among current smokers (Table 2). Adjusted ORs for SCC were much greater than those for AC. Female former smokers were only at significant elevated risk for SCC.

Table 3

**Table 3:** Mean age and smoking status by histological type and filter/nonfilter cigarette consumption among male current smokers

		**Cases**			
	**Type of smoking**	**Squamous cell carcinoma**	**Adenocarcinoma**	**All lung cancers**	**Controls**
Number (%)	Mixed	114 (83.2)	84 (61.3)	259 (72.8)	80 (49.4)
	Filter	23 (16.8)	53 (38.7)	97 (27.2)	82 (50.6)
					
Mean age	Mixed	67.2	65.5	66.6	63.6
	Filter	56.3	52.3	53.2	51.6
					
Mean number of cigarettes per day	Mixed	28.3	26.4	27.3	23.9
	Filter	34.5	32.1	32.6	28.6
					
Mean total duration	Mixed	47.0	45.3	46.5	43.7
	Filter	34.1	30.4	32.0	28.5
					
Mean duration of filtered cigarettes	Mixed	29.4	29.2	29.5	26.6
	Filter	34.1	30.4	32.0	28.5
Mean duration of nonfiltered cigarettes	Mixed	17.6	16.1	17.0	17.2
	Filter	—	—	—	—

Mixed= nonfilter-exclusive smoker or filter/nonfilter-mixed smoker; Filter= filter-exclusive smoker.

shows mean age and smoking status in terms of filter/nonfilter cigarette consumption among current male smokers by histological type. Lifelong nonfilter-exclusive smokers comprised 7.5% (39 of 518 men). Filter-exclusive smokers were much younger and consumed more cigarettes per day. Total duration of smoking among nonfilter or mixed cigarette smokers was substantially longer than that of filter smokers; this difference was largely due to the duration of nonfilter cigarette smoking among nonfilter or mixed smokers. Although duration of filter cigarette smoking showed less variation, smoking duration was slightly longer among filter-exclusive smokers. Men with SCC were older, had smoked for a longer duration and consumed more cigarettes per day than men with AC, for both filter and nonfilter users.

Table 4

**Table 4:** Adjusted odds ratios (ORs) for lung cancer by histological type according to filter/nonfilter cigarette consumption and age

		**Cases**								
	**Controls**	**Squamous cell carcinoma**			**Adenocarcinoma**			**All lung cancers**		
	**No.**	**No.**	**OR1**^a^ (95% CI)	**OR2**^b^ (95% CI)	**No.**	**OR1**^a^ (95% CI)	**OR2**^b^ (95% CI)	**No.**	**OR1**^a^(95% CI)	**OR2**^b^ (95% CI)
*All subjects*										
Mixed	80	114	1.00 (ref.)	1.00 (ref.)	84	1.00 (ref.)	1.00 (ref.)	259	1.00 (ref.)	1.00 (ref.)
Filter	82	23	0.52 (0.27–1.03)	0.55 (0.27–1.15)	53	0.88 (0.47–1.63)	0.83 (0.44–1.59)	97	0.70 (0.40–1.15)	0.70 (0.41–1.21)
										
*Age ⩽54*										
Mixed	12	5	1.00 (ref.)	1.00 (ref.)	4	1.00 (ref.)	1.00 (ref.)	13	1.00 (ref.)	1.00 (ref.)
Filter	53	8	0.38 (0.10–1.42)	0.38 (0.10–1.45)	33	2.01 (0.60–6.81)	2.54 (0.66–9.79)	54	1.00 (0.42–2.40)	1.05 (0.43–2.56)
										
*55 ⩽age ⩽64*										
Mixed	34	33	1.00 (ref.)	1.00 (ref.)	24	1.00 (ref.)	1.00 (ref.)	72	1.00 (ref.)	1.00 (ref.)
Filter	25	13	0.38 (0.10–1.42)	0.49 (0.18–1.35)	18	1.05 (0.46–2.43)	0.83 (0.33–2.11)	37	0.68 (0.34–1.36)	0.62 (0.29–1.35)
*Age ⩾65*										
Mixed	34	76	1.00 (ref.)	1.00 (ref.)	56	1.00 (ref.)	1.00 (ref.)	174	1.00 (ref.)	1.00 (ref.)
Filter	4	2	0.36 (0.05–2.24)	0.30 (0.05–1.97)	2	0.30 (0.05–1.74)	0.28 (0.05–1.71)	6	0.31 (0.08–1.21)	0.31 (0.08–1.20)

Mixed= nonfilter-exclusive smoker or filter/nonfilter-mixed smoker; Filter= filter-exclusive smoker.

a Adjusted ORs were presented after controlling for age and prefecture for all subjects, and for prefecture for each age-specific stratum.

b Additional control for number of cigarettes smoked per day.

shows adjusted ORs for lung cancer according to filter/nonfilter use among male current smokers by histological type. Overall, after adjustment for age and prefecture, OR for all lung cancers tended to be decreased by 30% (not significant) among filter-exclusive smokers as compared to mixed or nonfilter smokers. A nonsignificant tendency towards a reduction in adjusted OR was found for SCC, but not for AC. When we further examined the association between type of cigarettes and lung cancer histology according to age, by dividing participants into age groups of ⩽54-, 55–64-, and ⩾65-year old, ORs were shown to vary according to age and histology. Adjusted ORs in filter-exclusive smokers compared to mixed or nonfilter smokers decreased with increasing age group, regardless of histology. For men ⩽54-year old, a nonsignificant two-fold increase in risk in prefecture-adjusted OR of AC was observed among filter-exclusive smokers, whereas the adjusted OR indicated a nonsignificant 60% reduction in the risk of SCC. For men 55–64-year -old, a reduction in adjusted ORs in filter-exclusive smokers as compared to mixed or nonfilter smokers was observed for SCC, but not for AC. For the oldest group (⩾65-years), filter-exclusive smoking was associated with decreased risk irrespective of histological type. The reduction in adjusted ORs related to SCC and AC in filter-exclusive smokers was similar. Odds ratios after further controlling for mean number of cigarettes smoked per day were not substantially different, generally displaying a slight decline in adjusted ORs.

## DISCUSSION

The present study supports existing evidence of increased risks of both SCC and AC with higher numbers of cigarettes smoked and longer duration of smoking. In current smokers, risks indicated by adjusted ORs were higher for SCC than for AC. Furthermore, overall, although filter cigarette smokers were at lower risk compared to nonfilter smokers regardless of histology, a greater reduction in adjusted OR was observed for SCC than for AC.

Lower risk of all lung cancers has been observed among filter cigarette smokers compared to nonfilter cigarette smokers in some case–control studies of men (Wynder and Stellman, 1979; Lubin *et al*, 1984; Benhamou *et al*, 1989; Benhamou *et al*, 1994; Armadans *et al*, 1999) and women (Wynder and Stellman, 1979; Lubin *et al*, 1984; Agudo *et al*, 2000). However, the reduction in risk of all lung cancers among filter cigarette smokers compared to nonfilter cigarette smokers has been obscured. This results from the fact that the total incidence of lung cancer has increased in recent years, despite the widespread predominance of filter cigarettes. It is possible that, since this move towards filter cigarettes, insufficient time has elapsed to reflect a reduction in lung cancer incidence. Furthermore, overall lung cancer mortality rates had been increasing, although they have tended to level off in the last 5 years (Yoshimi *et al*, 2003). Separate analysis of an association between type of cigarette and lung cancer should therefore be performed by histological type of lung cancer.

One US case–control study has shown that the effect of filter cigarettes varies depending on the histological type of lung cancer, and revealed that reduced risk of SCC, but not AC, was apparent among filter cigarette smokers compared to nonfilter smokers (Stellman *et al*, 1997). These results are consistent with those of the present study. Another case–control study demonstrated a reduction in risk of Kreyberg I lung cancer, but not of Kreyberg II, among filter smokers (Wynder and Kabat, 1988). Trends towards a relative increase in AC compared to SCC might be partially attributable to a greater reduction in SCC among filter cigarette smokers compared to nonfilter cigarette smokers.

However, we cannot assume that the relative increase in AC observed in Japan is attributable to the same mechanisms seen in Western countries, since smoking has a lower impact on lung cancer risk among Asian populations and overall lung cancer death rates are lower in Japan than in Western countries (Sobue *et al*, 2002). This implies that factors other than smoking, such as lifestyle and diet, particularly the traditional Japanese diet, play important roles in lung cancer development (Wynder and Hoffmann, 1994). Furthermore, the association between certain dietary factors and lung cancer may be histological type specific (De Stefani *et al*, 1997; Kubik *et al*, 2001). The traditional Japanese diet, incorporating elements such as fish and soybean products, was found to be associated with a reduced risk of AC (Takezaki *et al*, 2001), and one study found the protective effects of tofu (a soybean product) appeared more significant for SCC (Wakai *et al*, 1999). In contrast, high levels of fat consumption increase the risk of lung cancer, particularly for AC (Ozasa *et al*, 2001). The Japanese diet has recently undergone substantial Westernisation, and such dietary alterations might represent an alternate explanation for the observed changes in histological types of lung cancer.

It should be noted that the effects of filter cigarettes on lung cancer by histological type varied according to age. Among men aged ⩽54 years, elevation in the adjusted OR for filter cigarettes compared to nonfilter cigarettes was found for AC, but not SCC. For men aged 55–64 years, use of filter cigarettes was associated with a reduction in adjusted ORs for SCC, but not for AC, whereas the magnitude of reduction was similar for SCC and AC in men ⩾65 years. No clear explanation for the age-related effects of filter cigarettes was apparent. Among young smokers, the tendency of filter smokers to inhale deeply to compensate for the low-tar delivery of filter cigarettes (Wynder and Muscat, 1995) might sufficiently affect the more peripheral regions of the lung, where most AC appear, even among short-term filter cigarette smokers. However, for older smokers, and considering long-term smoking, the cumulative exposure to tar contained in smoke might be substantially reduced among filter-exclusive cigarette smokers than among nonfilter or mixed cigarette smokers. The total protective effects for both SCC and AC might therefore be more apparent among older smokers. Indeed, trends in SCC and AC incidence from 1974 to 1997 among Japanese men differed according to age group, and the relative increase of AC compared to SCC was intensified in younger age groups (Yoshimi *et al*, 2003). Any elevation in risk of AC (or a smaller reduction in AC compared to SCC) attributable to filter cigarette use among younger smokers might represent an important issue, as the younger the age group, the more the smokers consume filter cigarettes in preference to nonfilter cigarettes. However, since no studies have addressed age-specific or duration-dependent protective effects of filter cigarettes, further confirmation is needed for other populations.

Reasons other than deep inhalation have been proposed as being responsible for filter cigarettes not providing relative protection against AC. These include reduced tar and nicotine delivery in filter cigarettes. Filter cigarettes remove the larger carcinogenic particles, meaning that smaller particles in the smoke from filter cigarettes reach the peripheral regions of the lung. Although tar delivery from filter cigarettes is reduced, concentrations of nitrosamines such as NNK (4-(methylnitrosamino)-1-3(pyridil)-1-butanone), which is known to induce the formation of AC, are not decreased in filter smoke (Agudo *et al*, 2000).

When we analysed the association between filter and nonfilter smoking, the most likely confounding factors were age and total duration of smoking. Duration of smoking was strongly associated with both SCC and AC in a dose-dependent manner. As age and total duration of smoking were also well correlated (correlation coefficient=0.80 (*P*<0.0001)), we avoided simultaneous inclusion of these variables in the logistic regression model. However, residual confounding related to cumulative smoking exposure might be partially responsible for the protective effects of filter cigarettes observed. In our subjects, mean number of cigarettes smoked per day was associated with risk of overall lung cancers and was higher among filter cigarette smokers than among nonfilter or mixed smokers. Comparison of filter/nonfilter cigarette smokers might thus be confounded by daily cigarette consumption. In this regard, the relationship between type of cigarette and lung cancer with adjustment for mean number of cigarettes is interesting. However, controlling for the amount of smoking, as a measure of exposure to lung carcinogens, requires caution when comparing the risks of different types of cigarettes. As low-nicotine low-tar filter cigarette smokers tend to smoke more cigarettes in order to maintain nicotine intake, adjustment by number of cigarettes may not be appropriate in case comparison between low-tar filter smokers and nonfilter or mixed cigarette smokers. These result in a spurious reduction in risk for filter cigarette smokers as compared to nonfilter or mixed cigarette smokers. Indeed, after adjustment for mean number of cigarettes per day, most adjusted ORs for overall lung cancers, SCC, and AC for filter cigarette smokers as compared to nonfilter or mixed cigarette smokers were slightly decreased. However, the increased risk of AC among men ⩽54-year and no reduction in the risk of AC among men aged 55–64 years were not changed even after controlling for this variable.

As controls were patients admitted to hospital, and controls with conditions known to be related to cigarette smoking were excluded, the possibility of underestimating lung cancer risks in smokers due to over-representation of smokers among hospital patients was minimised. However, one limitation of the present study should be considered. Smoking histories were obtained using self-reported questionnaires, and the number of subjects recording complete smoking histories was low; among current smokers, only 65% of the original subjects eligible as cases or controls were used for further analysis of associations between type of cigarette and lung cancer. The present study might therefore lack the power to detect slight to moderate increases (or decreases) in ORs. Finally, we did not obtain data on brand names of cigarettes smoked, which might have led to misclassification of cigarette types.

In conclusion, this study of a Japanese population revealed a type-specific association of filter cigarettes as compared to nonfilter cigarettes, that is, a protective effect against SCC, but no such effect (or, at least, a relatively reduced effect) against AC, particularly among younger smokers. Further confirmation is required to ascertain possible differences in risks of filter cigarettes for lung cancer. However, almost all smokers have now changed to filter cigarettes. The more prevalent smoking of filter cigarettes becomes, the more limited the opportunities for further investigations comparing filter and nonfilter cigarettes.
